# Structure of prothrombin in the closed form reveals new details on the mechanism of activation

**DOI:** 10.1038/s41598-018-21304-1

**Published:** 2018-02-13

**Authors:** Mathivanan Chinnaraj, Zhiwei Chen, Leslie A. Pelc, Zachary Grese, Dominika Bystranowska, Enrico Di Cera, Nicola Pozzi

**Affiliations:** 1 Edward A. Doisy Department of Biochemistry and Molecular Biology, Saint Louis University School of Medicine, St. Louis, MO 63104 USA; 2 Department of Biochemistry, Wroclaw University of Science and Technology, Wybrzeze Wyspianskiego 27, 50-370 Wroclaw, Poland

## Abstract

The clotting factor prothrombin exists in equilibrium between closed and open conformations, but the physiological role of these forms remains unclear. As for other allosteric proteins, elucidation of the linkage between molecular transitions and function is facilitated by reagents stabilized in each of the alternative conformations. The open form of prothrombin has been characterized structurally, but little is known about the architecture of the closed form that predominates in solution under physiological conditions. Using X-ray crystallography and single-molecule FRET, we characterize a prothrombin construct locked in the closed conformation through an engineered disulfide bond. The construct: (i) provides structural validation of the intramolecular collapse of kringle-1 onto the protease domain reported recently; (ii) documents the critical role of the linker connecting kringle-1 to kringle-2 in stabilizing the closed form; and (iii) reveals novel mechanisms to shift the equilibrium toward the open conformation. Together with functional studies, our findings define the role of closed and open conformations in the conversion of prothrombin to thrombin and establish a molecular framework for prothrombin activation that rationalizes existing phenotypes associated with prothrombin mutations and points to new strategies for therapeutic intervention.

## Introduction

Trypsin-like proteases and their zymogen precursors play dominant roles in enzyme cascades such as blood coagulation, complement and fibrinolysis where they often appear in complexes assembled on biological membranes^1,2^. The conversion of prothrombin (proT) to thrombin by the prothrombinase complex, composed of the enzyme factor Xa (fXa), the cofactor Va (fVa), Ca^2+^ and phospholipids, provides a biologically relevant example^3,4^. The reaction is essential for life and a key target of anticoagulant therapy^5,6^. Yet, its mechanism remains poorly understood in structural terms.

**Figure 1:**
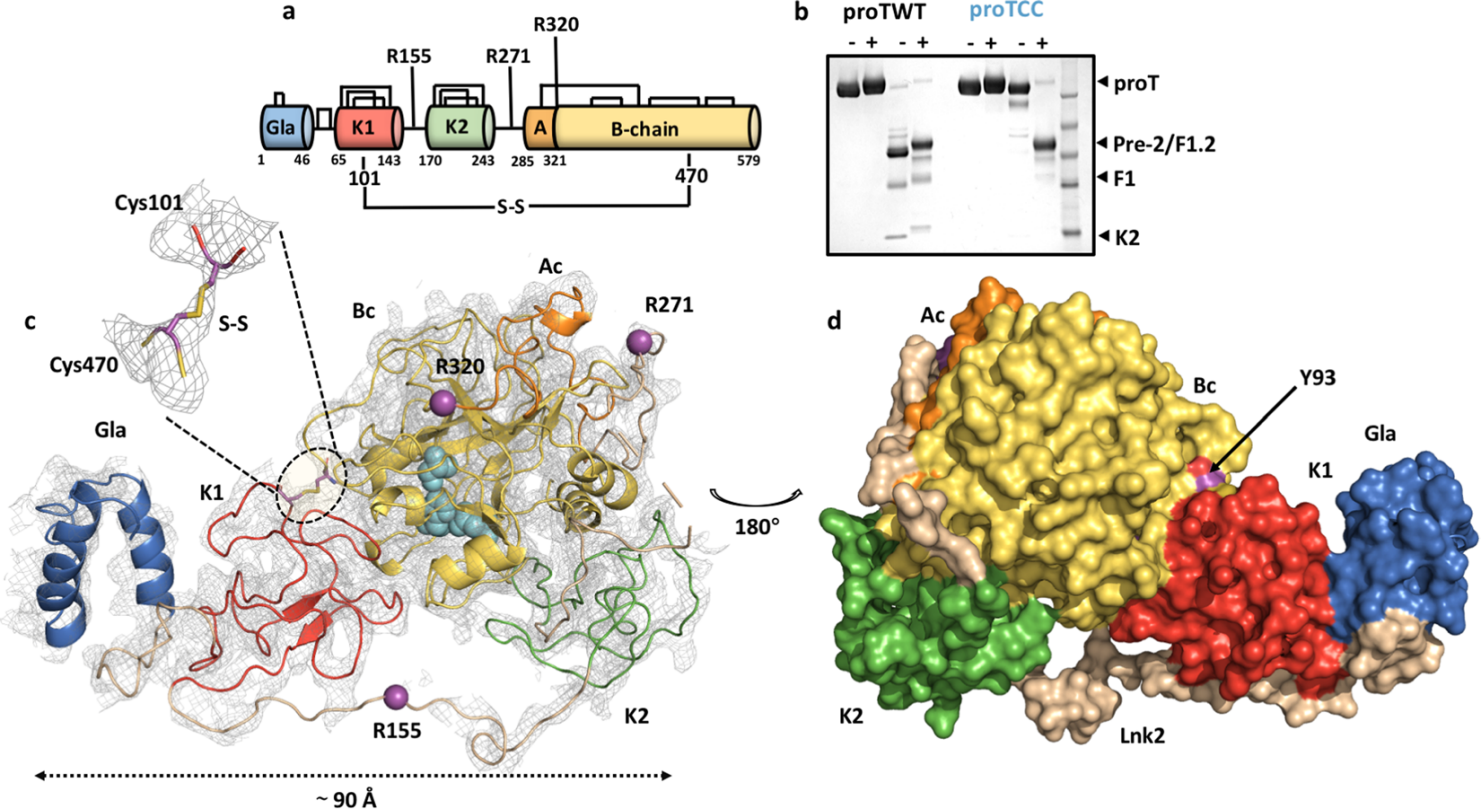
Engineering and overall structure of the closed conformation of prothrombin. (**a**) Color-coded domain architecture of prothrombin displaying the location of the engineered disulfide bond linking kringle-1 with the protease domain. Natural disulfide bonds are shown as black lines and positioning of the cleavage sites are indicated. (**b**) Limited proteolysis of protWT (lanes 0–4) and proTCC (lanes 5–8) by fXa (100 nM) in the absence (−) and presence (+) of reducing agent. Shown are times 0 (lanes 1–2 and 5–6, 6 μg) and 120 min (lanes 3–4 and 7–8, 2.5 μg). The MW marker is shown in lane 9 (62,49,38,28,17 kDa). The full-length gel is presented in Supplementary Fig. 5. (**c**) Overall structure of proTCC solved at 4.1 Å resolution colored as in a shown as cartoon (left) or surface (right) after 180° rotation. The key residue Tyr93 sits at the interface between kringle-1 and the protease domain. fXa cleavage sites Arg155, Arg271 and Arg320 are shown as spheres (magenta). The catalytic triad His363, Asp419 and Ser525 is shown as spheres (cyan). Zoom-in view of the artificial disulfide bond Cys101-Cys470. The electron density 2Fo-Fc map is countered at 1.5σ.

Prothrombin, or clotting factor II, is a modular protein composed of 579 amino acids organized into the Gla domain (residues 1–46), kringle-1 (residues 65–143), kringle-2 (residues 170–248), and the protease domain (residues 285–579) connected by three intervening linkers (Fig. 1a)^7,8^. Activation of prothrombin by prothrombinase involves cleavage at two distinct sites, Arg271 and Arg320, along two alternative pathways that generate the zymogen precursor prethrombin-2 and the active enzyme meizothrombin, respectively^3,8^. Although these intermediates do not accumulate under conditions relevant to physiology^9^, selection of the pathway of activation is of mechanistic interest because it defines the rate of thrombin generation depending on the context^10–13^.

Recent studies with prothrombin derivatives devoid of segments of Lnk2, the flexible linker connecting kringle-1 to kringle-2, have provided new insights into the mechanism of prothrombin activation^7,14^. A high-resolution crystal structure of the prothrombin mutant proTΔ154-167, lacking 14 residues of Lnk2, shows the domains of the zymogen vertically aligned along the main axis of the molecule. Interestingly, the mutant is activated by fXa in the presence of phospholipids at a rate >10-fold faster than wild-type (proTWT). The observation suggests that “shortening” of Lnk2 and stabilization of a conformation similar to that of proTΔ154-167 contributes to the 2,000-fold rate enhancement in prothrombin activation produced by cofactor fVa in prothrombinase. The hypothesis becomes relevant in the context of the recent observation that prothrombin exists in equilibrium between “open” and “closed” conformations that differ ~50 Å in overall length and with the closed form being predominant in solution (80%)^15^. The X-ray structure of proTΔ154-167 is a good representation of the open form, but the structural architecture of the closed form remains elusive. Single-molecule FRET (smFRET) measurements, mutagenesis and limited proteolysis suggest that the closed form features an intramolecular collapse of Tyr93 in kringle-1 onto Trp547 in the active site of the protease domain. Structural validation of this collapse is timely and important. Furthermore, constructs selectively stabilized in the open and closed forms may reveal the role of these distinct conformations in the mechanism of prothrombin activation.

In this study, we report the characterization of the prothrombin mutant proTS101C/A470C (proTCC) in which kringle-1 is linked to the protease domain by an engineered disulfide bond. X-ray crystallography and smFRET measurements prove that proTCC captures essential features of the closed form of prothrombin. Kinetic measurements with mutants selectively stabilized in the open and closed forms reveal how these forms contribute to the rate and pathway of prothrombin activation.

## Results

### Stabilization of the closed form by protein engineering

Recent smFRET studies indicate that probes attached to kringle-1 and the protease domain of prothrombin are in close proximity (<30 Å) in the closed form but widely separated in the open form^15^. This observation suggested that kringle-1 and the protease domain could be covalently linked in the closed form. Initial experiments with homobifunctional crosslinkers generated heterogeneous products not suitable for structural and functional studies because of lack of specificity of the NHS chemistry. Ser101 in kringle-1 and Ala470 in the protease domain (Fig. 1a) were then selected for Cys replacement. Ser101 was previously mutated to Cys for labeling and smFRET measurements and caused no detectable perturbations of function. Ala470 is located in the flexible autolysis loop widely exposed to solvent and far enough away from other Cys residues. The double mutant proTS101C/A470C (proTCC) expressed in high yield in mammalian cells and was purified to homogeneity. Presence of the disulfide bond was verified by limited proteolysis with fXa, which is the physiological activator of prothrombin (Fig. 1b). fXa cleaves proTWT at Arg271 (Fig. 1a) and generates fragment 1.2 (F1.2; 1–271) and prethrombin-2 (pre-2; 272–579) with similar electrophoretic mobility under non-reducing and reducing conditions (Fig. 1b, lanes 2–3). Cleavage of proTCC at Arg271 produces two fragments visible only under reducing conditions because of the presence of the disulfide bond between residues 101 and 470 (Fig. 1b, lanes 5–6).

### X-ray crystal structure of the closed form

Crystallization of prothrombin is complicated by the flexibility of linker regions like Lnk2^7^. Locking the conformational ensemble in the closed form with proTCC produced crystallization under several conditions in only four days, yet crystals diffracted poorly (to 6 Å) despite several rounds of optimization over 10+ months. Only one crystal from the same screen diffracted to higher resolution (4.1 Å) after growing for >2 months. In both cases, the structure could be solved by molecular replacement using the structural model of the closed form generated from recent smFRET measurements in which Lnk2 (154–167) and Lnk3 (257–275) were deleted due to their uncertain position (Table 1). Overall, proTCC spans ~90 Å in length (Fig. 1c,d), with the N-terminal Gla-domain, kringle-1 and kringle-2 aligned on a common axis and the protease domain folded back onto kringle-1 in an interaction that buries ~1253 Å^2^ of accessible surface area. The engineered disulfide bond Cys101-Cys470 is traceable in the electron density map (Fig. 1c) and so are the other 12 natural disulfide bonds (Fig. 1S).

**Table 1:** Crystallographic data for human prothrombin mutant S101C/A470C.

	Form I	Form II
Buffer/salt	100 mM Bicine, pH 8.5	100 mM Bicine, pH 9.0
PEG	3350 (16%)	3350 (11%)
PDB ID	6BJR	6C2W
**Data collection**	Raxis IV^++^	Raxis IV^++^
Wavelength (Å)	1.54	1.54
Space group	C222_1_	P1
Unit cell dimensions (Å)	a = 114.1, b = 124.2, c = 157.1	a = 85.0, b = 85.1, c = 153.8 α = 105.3°, β = 90.0°, γ = 90.1°
Molecules/asymmetric unit	1	2
Resolution range (Å)	40–6.0	40–4.1
Observations	23586	76078
Unique observations	2633	30018
Completeness (%)	87.6 (58.2)	91.7 (74.1)
R_sym_ (%)	18.0 (90.8)	15.6 (67.5)
I/σ(I)	9.3 (1.7)	5.7 (1.3)
**Refinement**		
Resolution (Å)	40–6.0	40–4.1
R_cryst_, R_free_	0.201, 0.289	0.278, 0.333
Reflections (working/test)	2357/268	26884/1394
Protein atoms	4594	9188
Rmsd bond lengths^a^ (Å)	0.010	0.009
Rmsd angles^a^ (°)	1.4	1.2
**Ramachandran plot**		
Most favored(%)	97.2	96.6
Generously allowed (%)	1.6	2.5
Disallowed (%)	1.2	0.9

^a^Root-mean-squared deviation (Rmsd) from ideal bond lengths and angles.

**Figure 2:**
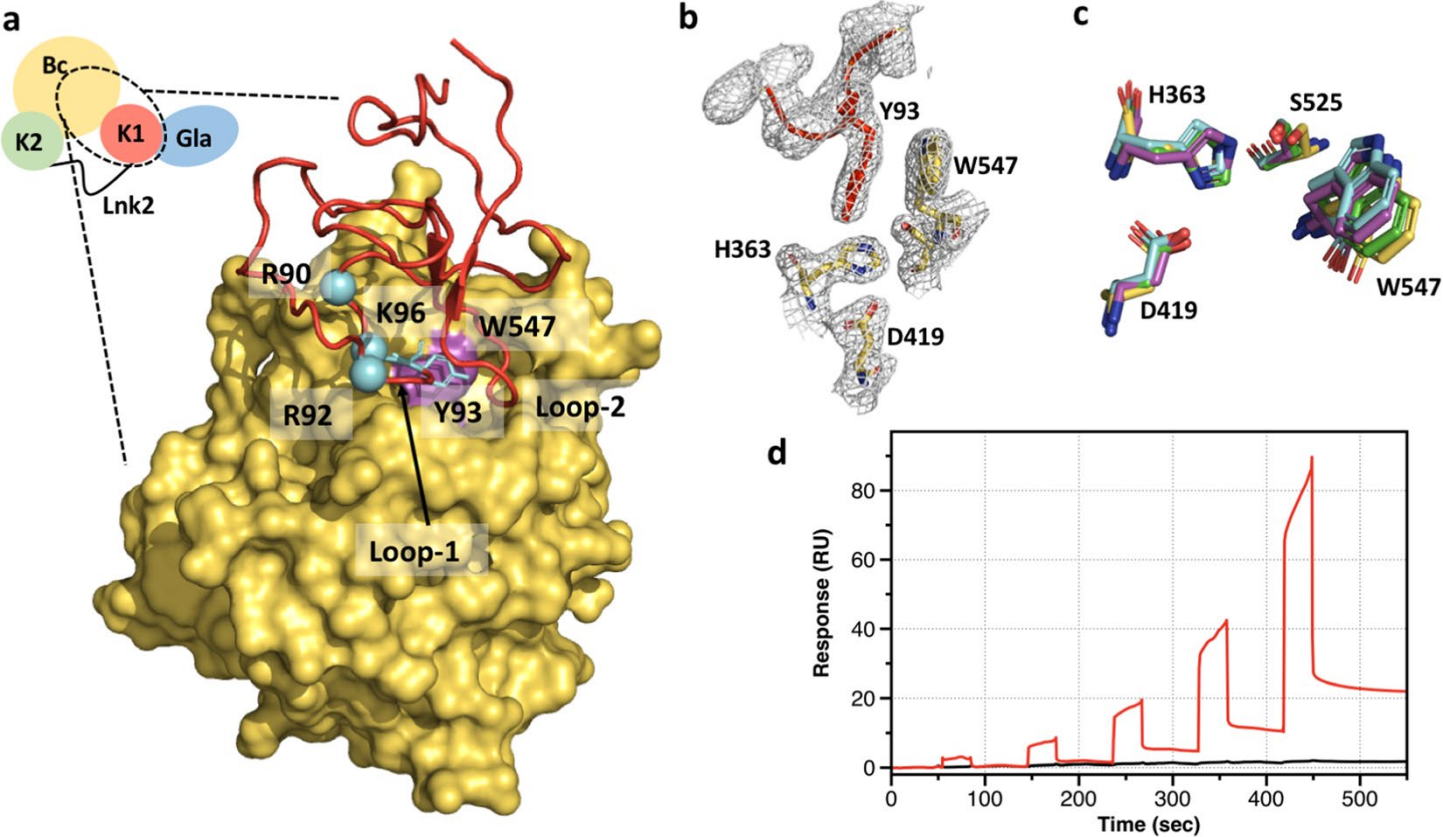
Intramolecular interactions between kringle-1 and the protease domain. (**a**) Zoom-in view of the interface between kringle-1 (cartoon, red) and the protease domain (surface, yellow); positively charged residues of loop-1 (Arg90, Arg92 and Lys96) are shown in cyan. Residue Tyr93 in kringle-1 is shown in stick representation while residue Trp547 is colored in magenta. (**b**) Zoom-in view of the face-to-face interaction between Tyr93 and Trp547 in the structure of proTΔ154-167 solved at 2.2 Å resolution^14^. The electron density 2Fo- Fc map is countered at 2.0σ. (**c**) Structural overlay of the catalytic triad (His363, Asp419, Ser525) and Trp547 of proTCC (yellow), proTΔ154-167 (5ED3^14^, cyan), proTΔ146-167 form-1 (4O03^7^, magenta) and proTΔ146-167 form-2 (4NZQ^14^, green). (**d**) Binding of fragment-1 to prethrombin-2 monitored by SPR. Two-fold dilutions of fragment-1, from 119 µM to 7.4 µM, were injected over both the active prethrombin-2 surface and an unmodified CM5 surface. Reference-subtracted data is shown in red. Buffer-only injections are shown for the purposes of comparison in black.

Two loops in kringle-1 (loop-1 composed of residues 90–94, and loop-2 composed of residues 130–135) contribute to the binding interface with the protease domain which is dominated by hydrophobic contacts (Fig. 2a) and facilitated by electrostatic complementarity between the positively charged edge of kringle-1 (Arg90, Arg92, Lys96) and the negatively charged loops surrounding the active site^16^. Prothrombin’s compact fold is sufficient to confirm the important role of Tyr93 inferred from previous smFRET measurements and to explain how perturbation of Tyr93 and its interactions destabilize the closed form. Tyr93 is located at the tip of loop 1 and inserts the aromatic side chain into a deep cavity of the protease domain where it engages Trp547 (Fig. 2a). Due to the low resolution of the structures, the binding interface could not be rendered in atomic details. However, we note that the same hydrophobic interaction (Fig. 2b) and a similar organization of the active site (Fig. 2c)^7,14^ are distinguishing elements of the crystal packing in all structures of prothrombin devoid of Lnk2.

Further evidence for the interaction between fragment-1 and the serine protease domain was obtained by Surface Plasmon Resonance (Fig. 2d). Fragment-1 binds to immobilized prethrombin-2 in a dose-dependent manner. In the absence of the linker connecting the two kringles, the interaction is weak (>200 μM) and complex, characterized by at least two dissociation rate constants. At the highest concentration tested (119 µM), only 83 RU of fragment-1 binds, with roughly 70% of the bound fragment-1 dissociating rapidly from prethrombin-2. However, roughly 30% of bound fragment-1 (25 RU) appears to dissociate slowly from prethrombin-2. Whether this kinetic profile is due to a two-state binding mechanism, dimerization of fragment-1 and/or heterogeneous binding due to change in prethrombin-2 during the coupling process will require further investigation.

**Figure 3:**
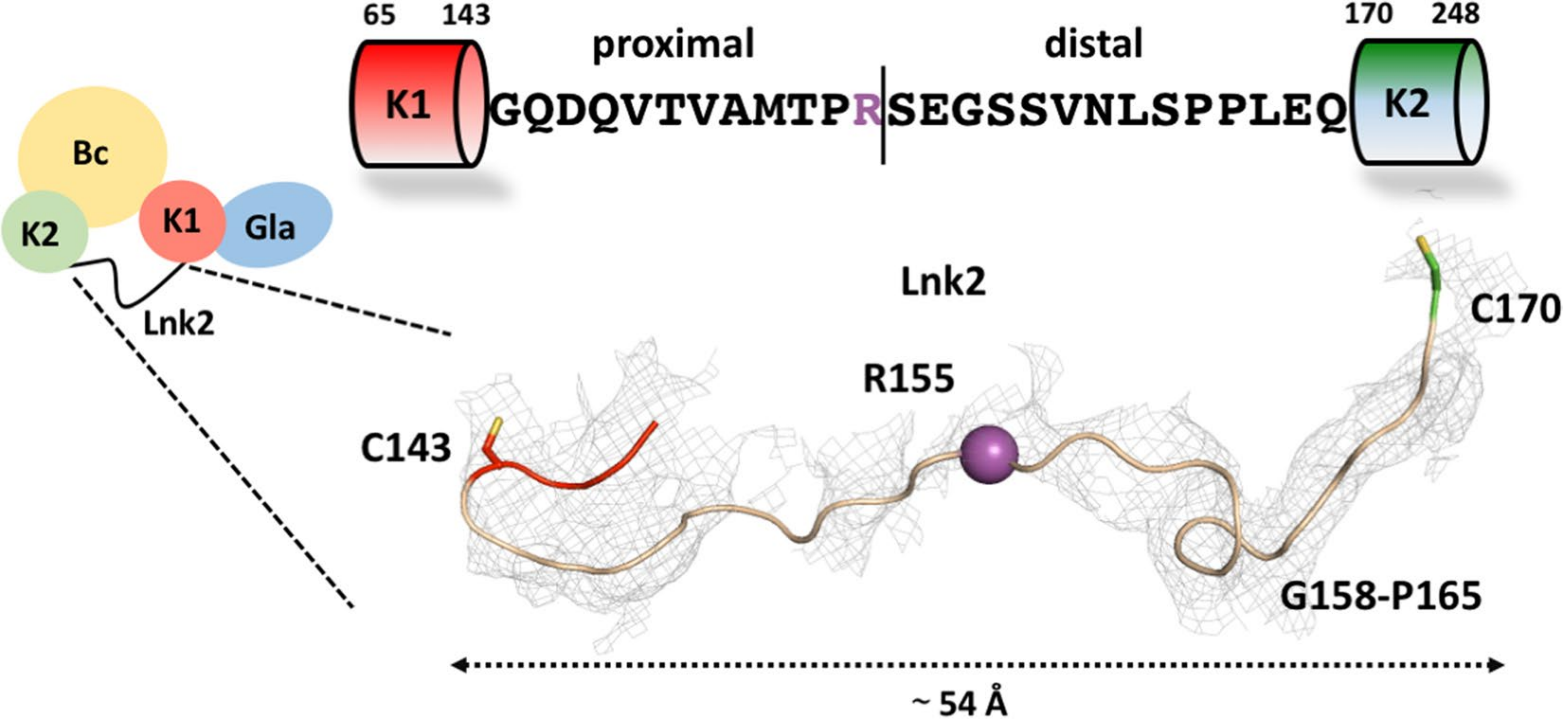
Primary sequence and zoom-in view of the Lnk2 connecting kringle-1 to kringle-2. Residues 149, 150 and 154 have weak electron density. The cleavage site Arg155 is shown as spheres (magenta). The electron density 2Fo-Fc map is countered at 1.0σ.

The rigidity of the closed form influences Lnk2 that appears in almost its entirety as judged by the presence of extra electron density map connecting the two kringles obtained from molecular replacement (Fig. 3 and Fig. S2). The highly conserved proximal portion of Lnk2 (Gly144-Arg155) runs close to kringle-1 and likely engages this domain in several H-bonding interactions, as seen in the high-resolution crystal structure of the mutant proTΔ154-167^14^. In the distal segment of Lnk2, residues 158–165 form a short helix turn before connecting to residue Cys170 in kringle-2. The helix explains why short deletions in this portion of Lnk2 may not alter the overall length of the connection between the two kringles and are functionally inconsequential. The calculated Cα-Cα distance between Cys143 in kringle-1 and Cys170 in kringle-2 is ~54 Å, implying that at least 15 residues are necessary to maintain prothrombin in the closed conformation and that fewer residues in Lnk2 would force the conformation to open. This is in very good agreement with recent functional data^7,14^.

### The closed form in solution

**Figure 4:**
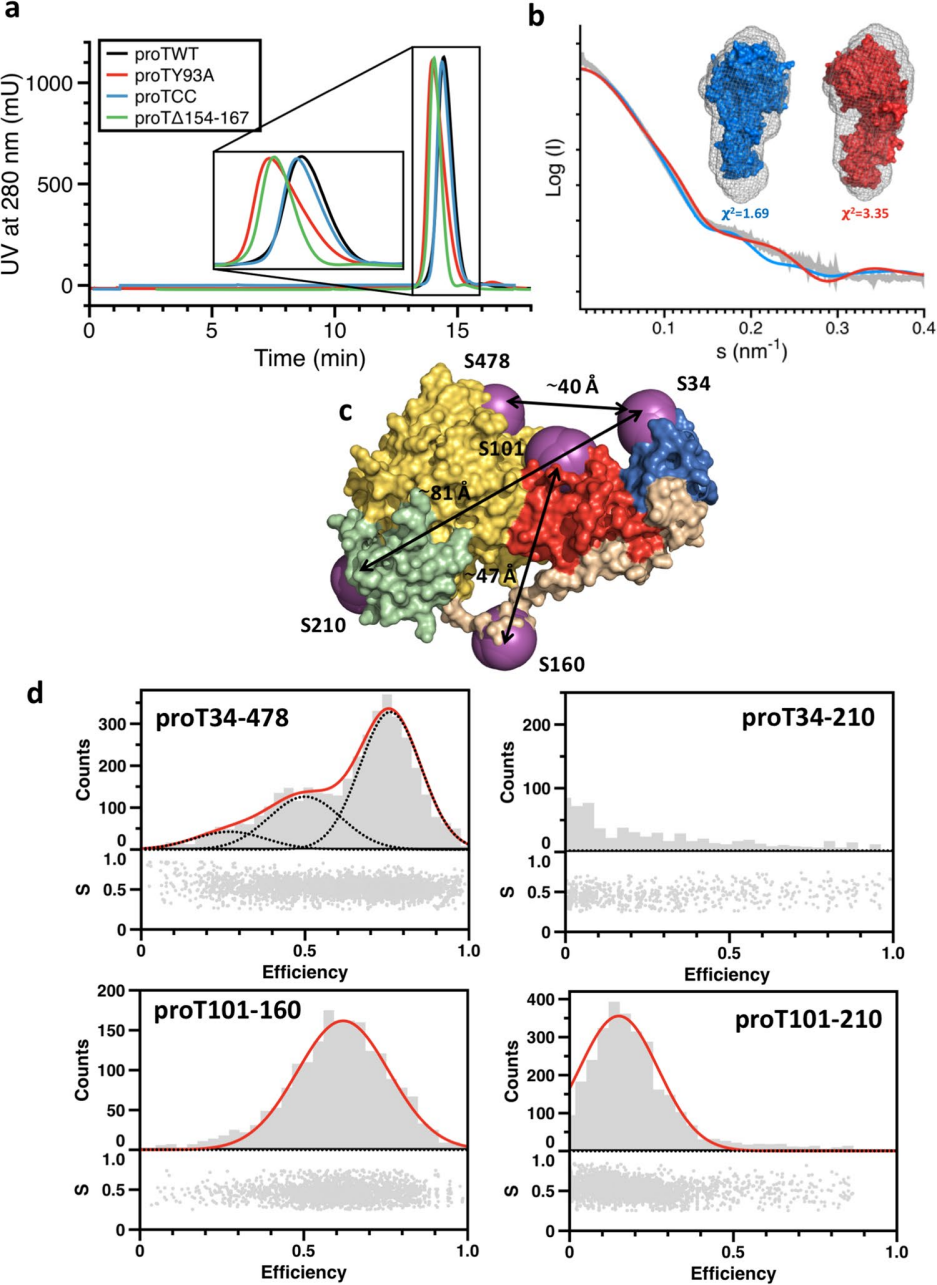
Solution structure of proTCC. (**a**) Elution profile of a solution (100 μl, 1 mg/ml) of proTWT (black line), proTCC (blue line), proTY93A (red line) and proTΔ154-167 (green line) injected onto a Superdex 200 HR 10/30 GL at 0.5 ml/min. Closed (proTWT and proTCC) and open (proTY93A and proTΔ154-167) conformations of prothrombin show a small but significant difference of the retention volume (~0.5 ml), in accordance with their shape in solution. (**b**) SAXS profile and reconstructed 3D envelope of proTWT (gray) fit with the structure of the closed (protCC, red) or open (proTΔ154-167, blue) conformations of prothrombin. (**c**) Guided by the structure of protCC, smFRET pairs were designed to cover all possible domain combinations. Probes were attached at residues 34, 101, 120, 160, 210 and 478, which are shown are magenta spheres. Black arrows indicate Cα-Cα distances for 34/478, 34/210 and 101/160, as representative examples. (**d**) smFRET histograms for proT34/478, proT34/210, proT101/160 and proT101/210. The bottom section of the top graph of each construct depicts the stoichiometry, S, versus FRET efficiency for each diffusing molecule that contains both AF555 and AF647 fluorophores. The upper section shows the one-dimensional efficiency histogram of the molecules in the bottom section. Populations were fit to a single (101/160 and 101/210 FRET couples) or triple (34/478 FRET couple) Gaussian distribution (red lines).

The X-ray crystal structure of the closed form was validated by size exclusion chromatography (SEC), small angle x-ray scattering (SAXS) and smFRET of prothrombin in solution. SEC and SAXS inform on the hydrodynamic radius of a protein in solution, which is dictated by its shape. As such, the open conformation of prothrombin (proTY93A and proTΔ154-167) displays a significant shorter retention time compared to proTWT and proTCC, which is indicative of a more elongated shape (Fig. 4a). Likewise, the SAXS envelope reported recently for protWT^15^ fits significantly better to the structure of the closed conformation (proTCC) of prothrombin compared to the open form (proTΔ154-167)^14^ (Fig. 4b), suggesting that proTCC is the dominant conformation of prothrombin in solution.

We next performed smFRET experiments to confirm the structural similarities between proTCC and proTWT in solution. In addition to the previously reported FRET pairs 34/101, 101/478, 120/478, 210/478^15^, we generated four new diagnostic pairs 34/478, 34/210, 101/210, 101/160 to monitor signals across Gla domain, kringles and protease domain (Fig. 4c,d). Measurements were carried out on freely diffusing molecules using a confocal setup equipped with ns alternate excitation scheme to sort subpopulations of molecules according to stoichiometry^15^. All FRET pairs reported a dominant population consistent with the prevalence of the closed conformation in solution. FRET histograms were fit to Gaussian functions to extract the center of each distribution and the resulting values were compared to the average FRET efficiency obtained by simulations of fluorescent dyes attached at the mutated sites through flexible linkers (Tables 2, S3)^15,17^. The experimental values are fully consistent with the structural model of proTCC and support the structure of this mutant as a genuine representation of the closed form of prothrombin in solution.

**Table 2:** Comparison between experimental (Exp) and theoretical (Theo) FRET values.

FRET mutant	Domains	Exp	Theo	ΔFRET (Exp-Theo)
34–101	Gla-K1	0.94	0.92	0.02
34–210	Gla-K2	0	0.02	0.02
34–478	Gla-Bc	0.77	0.74	0.03
101–160	K1-Lnk2	0.62	0.23	0.39
101–210	K1-K2	0.15	0.11	0.04
101–478	K1-Bc	0.89	0.94	0.05
120–478	K1-Bc	0.58	0.55	0.03
210–478	K2-Bc	0.19	0.12	0.07

Standard deviations for experimental FRET values are typically 10%. Significant changes between experimental and theoretical values (>0.1) were observed only for the couple 101/160, indicating that the position of residue 160 in Lnk2 varies in solution and, on average, is closer to residue 101.

### Activation of prothrombin and the conformation of meizothrombin

**Figure 5:**
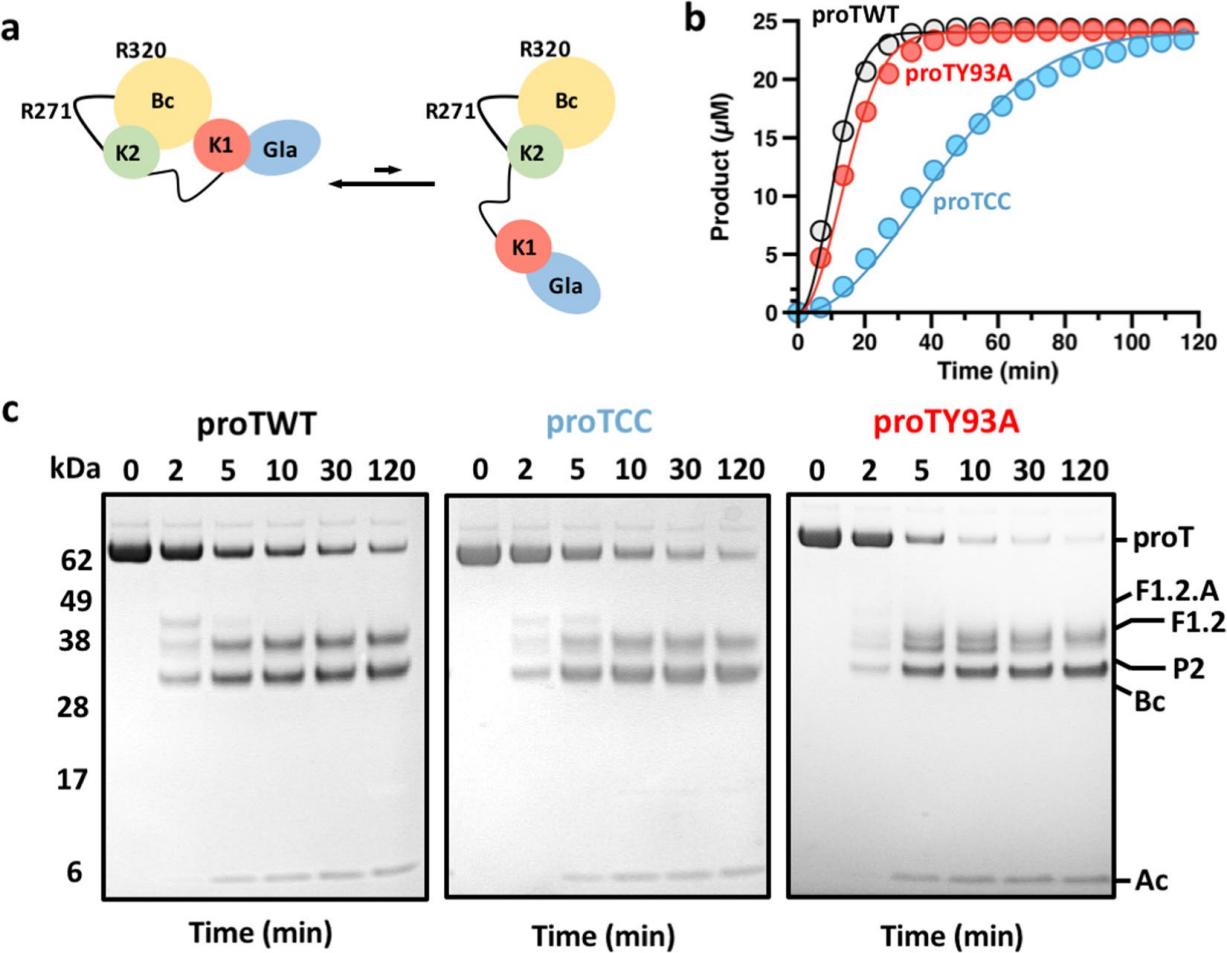
Kinetics of activation of open and closed conformations of prothrombin by the prothrombinase complex. (**a**) Graphical representation of the equilibrium between closed and open conformations of prothrombin that is shifted toward the closed form (>80%) under physiological conditions. ProTCC irreversibly locks prothrombin in the closed form. The mutation Y93A shifts the equilibrium toward the open form. (**b**,**c**) Activation of closed and open conformations of prothrombin by the prothrombinase complex monitored by chromogenic assay (**b**) or SDS-PAGE (**c**). Proteolytic fragments are indicated: prothrombin (proT), fragment-1.2.A (F1.2.A), fragment-1.2 (F1.2), prethrombin-2 (P2), B-chain (Bc), A-chain (Ac). Full-length gels are presented in Supplementary Fig. 5. Each measurement in panel b is the average of three individual determinations.

The availability of reagents stabilized in the open (proTY93A) and closed (proTCC) conformations enables direct assessment of the contribution of these forms to the rate and pathway of prothrombin activation (Fig. 5). The rate of conversion of prothrombin to thrombin was first measured by using a colorimetric assay that continuously reports the amount of thrombin generated by the prothrombinase complex^14^. Figure 5b shows the progress curves of substrate hydrolysis for proTWT, proTCC, and proTY93A and proTWT displays the fastest activation rate with a specificity constant *k*_*cat*_/K_M_ = 3.8 ± 0.2 × 10^8^ M^−1^s^−1^, in agreement with previous results^14^, followed by proTY93A with *k*_*cat*_/K_M_ = 2.2 ± 0.2 × 10^8^ M^−1^s^−1^. The ~1.5-difference between proTWT and proTY93A is small but statistically significant, and similar to the reduction observed with mutants devoid of Lnk2 that stabilize prothrombin in the open conformation^14^. On the other hand, the defect of proTCC is not due to a slower generation of thrombin (as demonstrated in the gel assay in Fig. 5c) but instead to the impaired catalytic efficiency of the resulting enzyme toward the chromogenic substrate FPF-pNA due to fragment-1 remaining covalently attached to the autolysis loop.

Additional information on the rate and pathway of prothrombin activation was obtained by a discontinuous kinetic assay monitored by gel electrophoresis (Fig. 5c). In this assay, prothrombinase cleaves prothrombin first at Arg320 to produce fragment-1.2.A (F1.2.A; residues 1–320), which appears as a faint band at 40 kDa, and a steady accumulation of the B-chain of thrombin (Bc; residues 321–579) that migrates at 30 kDa under reducing conditions. A following cleavage at Arg271 generates the A-chain (Ac; residues 272–320, 6 kDa) and fragment 1.2 (F1.2; residues 1–271, 36 kDa). The alternative pathway of activation starts with cleavage at Arg271 to generate fragment 1.2 (F1.2; residues 1–271) and prethrombin-2 (pre-2; residues 272–579) with comparable hydrodynamic radii under reducing conditions, but slightly different electrophoretic mobility. The second cleavage at Arg320 splits the A- and B- chains under reducing conditions. The prothrombin band disappeared at similar rates for proTWT and proTCC, but slightly faster for proTY93A (Fig. 5c). Importantly, activation of proTCC and proTY93A follows distinct activation pathways: proTCC is cleaved first at Arg320 like proTWT along the meizothrombin pathway, but proTY93A is cleaved first at Arg271 along the prethrombin-2 pathway. Hence, the choice of activation pathway is dictated by the specific conformation of prothrombin, open or closed. The dominant closed conformation is cleaved preferentially at Arg320 and the open conformation is cleaved preferentially at Arg271.

**Figure 6:**
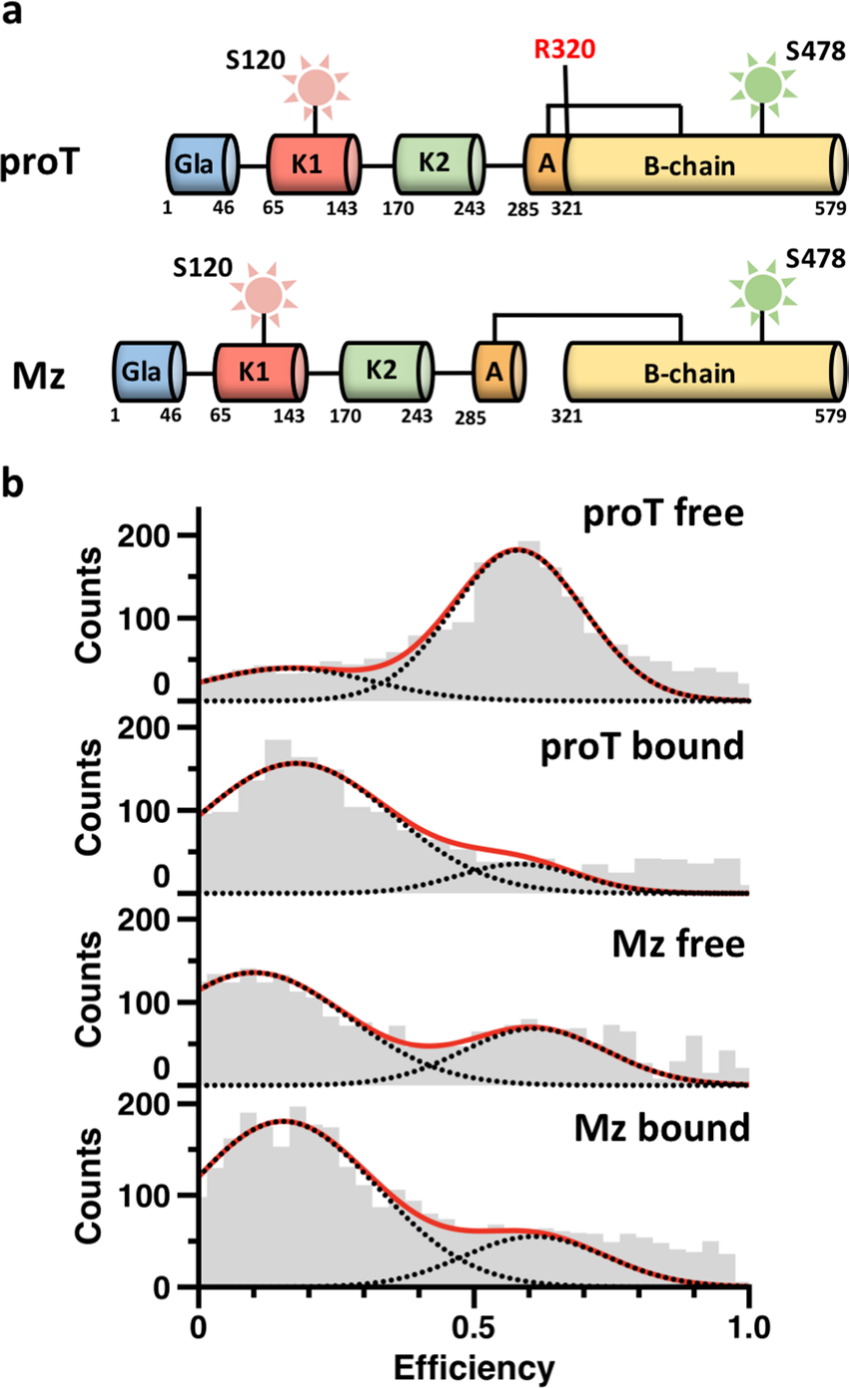
Structural architecture of meizothrombin in solution revealed by smFRET. (**a**) Cleavage of prothrombin (proT) at Arg320 generates meizothrombin (Mz). Fluorescent dyes were incorporated at positions S478 and S120, across the flexible Lnk2. (**b**) One-dimensional smFRET efficiency histogram for proT120/478/525 A and Mz120/478/S525A in the absence (free) or presence (bound) of argatroban (100 μM). Populations were fit to a double Gaussian distribution (red lines).

This observation invites attention on the conformation of the active intermediate meizothrombin, generated from prothrombin upon cleavage at Arg320 and presenting Arg271 for a second cleavage by prothrombinase to produce the mature enzyme thrombin. Meizothrombin retains the modular assembly of prothrombin (Fig. 6a) and may retain the conformational flexibility of the zymogen. The hypothesis was tested with smFRET measurements with the 120/478 pair monitoring the distance between kringle-1 and the protease domain across the flexible Lnk2 (Fig. 6b). Prothrombin shows two main distributions with the high FRET (E = 0.58) population (closed form) being dominant, in agreement with recent results^15^. Binding of argatroban shifts the equilibrium in favor of the low FRET (E = 0.16) population (open form). Meizothrombin also shows two main distributions but with the low FRET population (E = 0.12) being dominant and increased further by binding of argatroban. We conclude that meizothrombin exists in equilibrium between open and closed forms like prothrombin, but with the open conformation being preferred as seen for the prothrombin mutant Y93A.

## Discussion

The results reported in this study offer new insight into the mechanism of prothrombin activation under conditions relevant to physiology. In the blood, prothrombin circulates in equilibrium between closed and open conformations and binding of prothrombin to prothrombinase occurs primarily in the closed form, which is the conformation that predominates in solution. The closed form promotes cleavage at Arg320 along the meizothrombin pathway and the open form promotes cleavage at Arg271 along the prethrombin-2 pathway. This functional distinction extends to meizothrombin that exists predominantly in the open form and can only be cleaved at Arg271. We conclude that prothrombin binds to prothrombinase in the closed form and after cleavage at Arg320 switches to meizothrombin in the open form, which is then cleaved at Arg271 to produce the mature enzyme thrombin.

**Figure 7:**
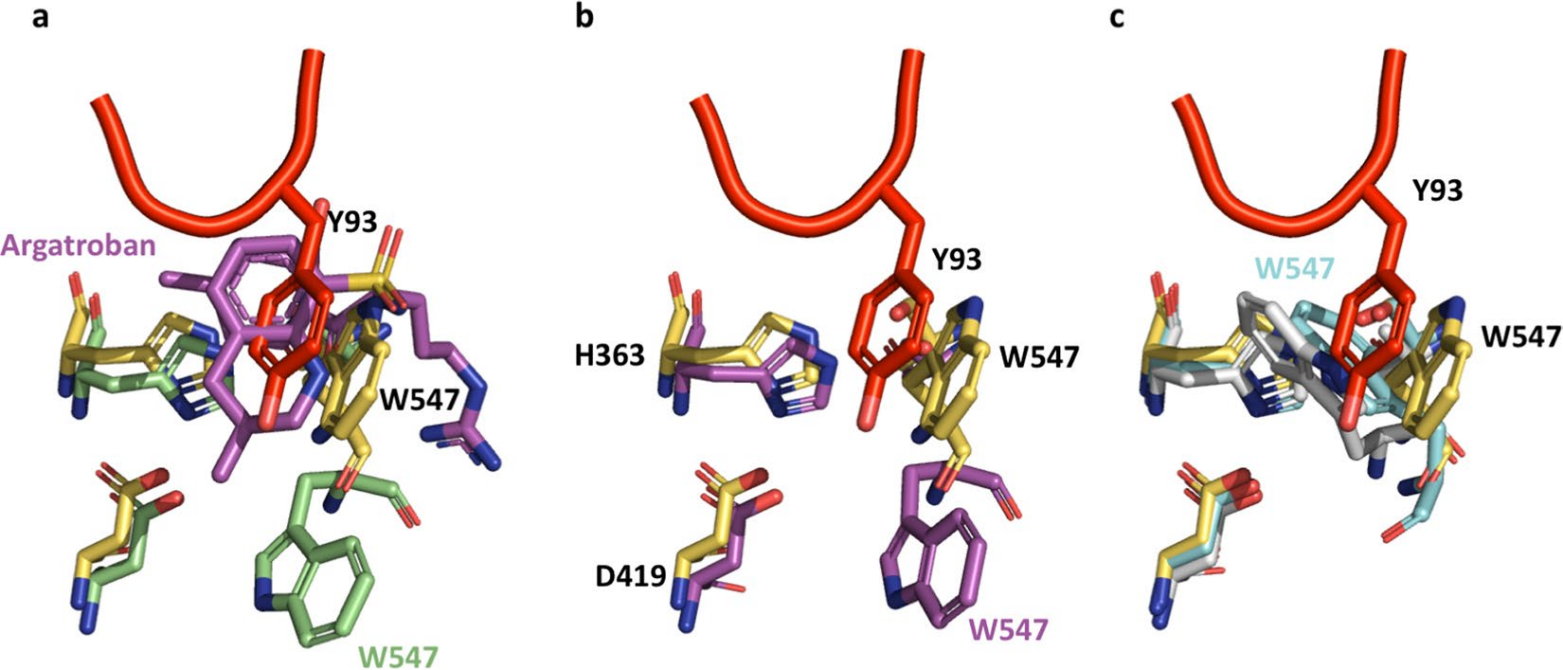
Mechanisms to destabilize the intramolecular interaction between kringle-1 and the protease domain thereby shifting the equilibrium toward the open form. (**a**) In prothrombin, Trp547 (5EDM, yellow stick) forms a face-to-face interaction with the aromatic side chain of Tyr93 (red stick). In the structure of the Na^+^-bound thrombin^27^ (1SG8, magenta stick) Trp547 relocates promoting opening of the active site. (**b**) Binding of argatroban (magenta stick) into the active site of prethrombin-2^34^ (4RN6, green stick) requires relocation of both Tyr93 and Trp547 (**c**) In the structure of prethrombin-1^30^ (3NXP, cyan stick) and prethrombin-2^23^ (3SQE, gray stick), Trp547 further collapses into the active site.

The foregoing mechanism explains why mutations that stabilize the open form of prothrombin, such as deleting >15 residues from Lnk2, substituting Tyr93 with Ala or occluding the active site (Fig. 7a), switch the pathway of activation from meizothrombin to prethrombin-2^7,14,18^. It also explains the slightly faster rate of thrombin generation in the chromogenic assays observed for proTWT compared to proTY93A and prothrombin mutants devoid of Lnk2 (Fig. 5b), in which cleavage occurs at Arg271 first resulting in accumulation of the inactive intermediate prethrombin-2. Interestingly, naturally occurring mutations Ala362Thr (Vellore 1)^19^, Glu466Ala (Salakta)^20^, and Gly548Ala (Perija)^21^ associated with a mild bleeding cluster near the interface between kringle-1 and the protease domain (Fig. 4S) and may perturb the closed-open conformational equilibrium.

Molecular details of the conformational transition between closed and open forms of prothrombin are provided by the X-ray crystal structure of the mutant proTCC. In this structure, prothrombin folds into an “L-shaped” form in which kringle-1 and protease domains engage in extensive intramolecular interactions. The domain-domain binding interface is dominated by the insertion of the aromatic side chain of residue Tyr93 in kringle-1 in the active site of the protease domain. One of the residues contributing to the interaction within this pocket is Trp547, which is known to exist in alternative conformations in the trypsin fold^22–24^ and likely influences the transition of prothrombin from the closed to the open conformation. Upon complex formation, cleavage of prothrombin at Arg320 generates a mature protease by reorganizing the active site, oxyanion hole and primary specificity pocket^25–27^ but also destabilizes the intramolecular collapse between kringle-1 and the protease domain most likely by relocating Trp547 (Fig. 7b). Cleavage of prothrombin at Arg320 promotes opening of the structure in solution, as documented by smFRET measurements of meizothrombin (Fig. 7b). However, when bound to prothrombinase, meizothrombin likely remains in a closed-like conformation and presents Arg271 to the active site of fXa. This sequence of events is consistent with the kinetic profile of the mutant proTCC, which is cleaved by the prothrombinase complex at Arg320 (first) and Arg271 (second) despite the presence of the disulfide bond that impedes elongation of the structure after cleavage at Arg320. We conclude that the role of fVa is to maintain prothrombin in a closed-like conformation by adding constraints on the structure that would otherwise switch to the open form after cleavage at Arg320. Presenting Arg271 to the active site of fXa may involve twisting of the protease domain on top of fragment-1, that remains anchored to the membranes^28,29^. Further investigation is necessary to test this hypothesis.

Cleavage at Arg320 is one of other mechanisms exploited by the trypsin-fold to promote opening of the prothrombin structure. Residue Trp547 is very flexible and completely occludes the active site pocket in the crystal structures of prethrombin-1^30^ and prethrombin-2^23^ (Fig. 7c). The collapsed conformation of Trp547 leaves no room for the side chain of Tyr93 and is found only in the structures of prethrombin-1 and prethrombin-2, that are intermediates along the pathway of prothrombin activation. It is possible that the active site of prothrombin changes its conformation upon cleavage at Arg155 generating prethrombin-1 or at Arg271 generating prethrombin-2. This would also explain why cleavage of proWT at Arg155 by thrombin liberates prethrombin-1 from fragment-1 without requiring cleavage at Arg320^15,31^.

The findings reported here also offer translational opportunities. Molecules that target the active site pocket of the zymogen would compete with Tyr93, stabilize the open form, drive activation toward the prethrombin-2 pathway and decrease the rate of thrombin generation. Indeed, activation of prothrombin mutants locked in the open form causes accumulation of prethrombin-2 and 1.7-fold prolongation of the activated partial thromboplastin time (aPTT) in human plasma^14^. Potential benefits of this class of inhibitors are safer pharmacological profiles (i.e., less bleeding complications) due to the modulation of thrombin production rather than direct inhibition of the mature enzyme^32^. Alternatively, stabilization of the open form via stalling of the prothrombinase complex after cleavage at Arg320 and generation of meizothrombin would afford an anticoagulant effect *in vivo*, as documented by the severe bleeding phenotype associated with the naturally occurring mutation prothrombin Padua, Arg271His, that abrogates cleavage site at Arg271^33^.

## Material and Methods

### Protein production and purification

Prothrombin cDNA wild-type (residues 1–579) modified to include an epitope for the HPC4 antibody at the C-terminal was cloned into a pDEST40 expression vector using the Gateway® cloning technology (Life Technologies, Carlsbad, CA)^14^. The mutations S34C, Y93A, S101C, S120C, S160C, S210C, S478C, A470C, and S525A were generated using the Quickchange Lighting kit (Agilent, Santa Clara, CA) and appropriate primers (Integrated DNA Technologies, Coralville, IA). After sequencing, the recombinant proteins proTWT, proT101/470, protY93A, proT34/101, proT34/210, proT34/478, proT101/160, proT101/210, proT101/478, proT120/478, proT210/478, proT120/478/S525A were expressed in BHK cells and purified by affinity chromatography, ion exchange chromatography, and size exclusion chromatography (SEC) as described previously^15^. SDS-PAGE and N-terminal sequencing verified homogeneity and chemical identity of final preparations. Activation of proT120/478/S525A (0.5 mg/ml) to Mz120/478/S525A was obtained with the snake venom ecarin (50 nM) for 3 hr at room temperature and the product was purified using ion exchange, and size exclusion chromatography before labeling. The mutation of the catalytic serine 525 was necessary to avoid autoproteolytic degradation of meizothrombin^34^. Human recombinant prethrombin-2 was expressed and purified as described elsewhere^23^. Small unilamellar vesicles composed of phosphatidylcholine (PC 100) or phosphatidylcholine and phosphatidylserine in a 3:1 molar ratio (PC:PS 75:25) were prepared by extrusion using 100 nm polycarbonate membranes (Avanti Polar Lipids, Alabaster, AL) and their size was confirmed by Dynamic Light Scattering (DLS). The vesicles were kept a 4 °C and used within 2 weeks. Protein concentrations were determined by reading at 280 nm with molar extinction coefficients adjusted based on the amino acid sequence. Human factor Xa, factor Va, ecarin, dansylarginine-N-(3-ethyl-1,5-pentanediyl)amine (DAPA) and fragment-1 were purchased from Hematological Technologies (VT). The chromogenic substrate H-D-Phe-Pro-Phe-p-nitroanilide (FPF-pNA) was from Midwest Bio-Tech (IN). All other chemicals were purchased from Sigma-Aldrich (MO).

### X-ray studies

Crystallization of the prothrombin mutant S101C/A470C carrying an artificial disulfide bond between the kringle-1 and the serine protease domain was achieved at 4 °C by the vapor diffusion technique, using the Art Robbins Instruments PhoenixTM liquid handling robot and mixing equal volumes of protein (10 mg/ml, 0.2 μl) and reservoir solution. Optimization of crystal growth was achieved by the hanging drop vapor diffusion method mixing 2–3 μl of protein (9.5–10.5 mg/ml) with equal volumes of reservoir solution. Crystals with size ~0.40 × 0.40 × 0.01 mm^3^ were grown in about one week at 4 °C in 100 mM bicine, pH 8.5 and 16% PEG 3350 for the 6 Å resolution structure. Crystals with size ~0.40 × 0.40 × 0.05 mm^3^ were grown in more than two months at 4 °C in 100 mM bicine, pH 9.0 and 11% PEG 3350 for the 4.1 Å resolution structure. Crystals were cryoprotected prior to flash freezing in a solution of 20% glycerol from the original mother liquor. X-ray diffraction data were collected with a home source (Rigaku 1.2 kW MMX007 generator with VHF optics) Rigaku Raxis IV++ detector and were indexed, integrated, and scaled with the HKL2000 software package^35^. Crystals showed anisotropic diffraction with 3.8 Å resolution in one direction and 6 Å resolution in another direction and 3.8 Å resolution in one direction and 4.5 Å resolution in another direction for the structures at 6.0 Å and 4.1 Å resolution, respectively. Several dehydration methods were tried for improving crystal diffraction^36^. Initially, the previous crystal structure of proTΔ154-167 solved at 2.2 Å resolution (Protein Data Bank code 5EDM)^14^ was used as search model using PHASER from the CCP4 suite^37^, but no solution was found because of a different orientation of fragment 1 in the new structures. Then molecular replacement was performed using the model previously built using smFRET data as a search model after energy minimization^15^ or using the structure of proTΔ154-167 partitioned into fragment 1 (1–153) and prethrombin-1 (168–579), and the two separate portions were used as individual search models using PHASER. The structures of linker 2 and residues 266–275 were traced based on the extra electron density obtained from molecular replacement. Refinement and electron density generation were performed with REFMAC5 from the CCP4 suite. 10% and 5% of the reflections were randomly selected as a test set for cross-validation for the structures at 6.0 Å and 4.1 Å resolution, respectively. For the structure at 4.1 Å resolution, twinned crystals belong to the space group P1 with cell dimensions a = 85.0 Å, b = 85.1 Å, c = 153.8 Å, α = 105.3°, β = 90.0° and γ = 90.1°, and the refinement was performed by twin laws. Model building and analysis of the structures were conducted with COOT^38^. Statistics for data collection and refinement are summarized in Table 1S. Atomic coordinates and structure factors have been deposited in the Protein Data Bank (accession codes: 6BJR for 6.0 Å resolution and 6C2W for 4.1 Å resolution). Structures at 6.0 Å and 4.1 Å resolution are superimposable.

### Surface Plasmon Resonance (SPR)

Human recombinant prethrombin-2 (200 μl, 30 µg/ml) was coupled to a Sensor Chip CM5 on a Biacore S200 (GE, USA) by N-hydroxysuccinimide (NHS)/1-Ethyl-3-[3-dimethylaminopropyl]carbodiimide hydrochloride (EDC) chemistry to reach a final ligand density of 1633 RU. The decision of immobilizing prethrombin-2 and not fragment-1 was based on the observation that prethrombin-2 showed significant more nonspecific binding compared to fragment-1. After initial binding tests to confirm interactions between fragment-1 and prethrombin-2, a single-cycle kinetic assay was set up to examine dose-response dependent. Increasing concentrations of fragment-1 (0–119 μM) were injected at a flow rate of 35 μl/min in 20 mM HEPES, 150 mM NaCl, 5 mM CaCl_2_, pH 7.4.

### SAXS

SAXS data were previously collected at the beamline 12-ID-B of the Advanced Photon Source at Argonne National Laboratory (Argonne, IL) on protWT (0.5–5 mg/ml)^15^. The low-resolution envelope was produced using both GASBOR (q up to 0.8 Å^−1^)^39^ and DAMMIN (q up to 0.3 Å^−1^)^40^ by directly fitting the reciprocal space scattering profile. Twenty models were generated for every calculation and then aligned and averaged using DAMAVER. Computing of the theoretical scattering profile of proTCC and proTΔ154-167 and fitting of the experimental profile was performed using the webserver FoXS^41^.

### Single-molecule FRET measurements of freely diffusing molecules

Selective labeling of the unpaired Cys residues with Alexa Fluor 555-C2-maleimide (AF555) as the donor and Alexa Fluor 647-C2-maleimide (AF647) as the acceptor (Thermo Fisher Scientific) was achieved and probed by limited proteolysis with thrombin, as described recently^15^. FRET measurements of freely diffusing single molecules were performed with a confocal microscope MicroTime 200 (PicoQuant, Berlin, Germany), as detailed elsewhere. Briefly, experiments were carried out with pulsed interleaved excitation (PIE)^42^, which reports the status of both donor and acceptor fluorophores by sorting molecules on the basis of relative donor:acceptor stoichiometry (S) and apparent FRET efficiency (E). The donor and acceptor dyes were excited with a ps pulsed diode laser at 532 and 638 nm, respectively. To achieve pulsed interleaved excitation, the 532 nm laser was electronically delayed 25 ns relative to the 638 nm laser. A dual band dichroic mirror reflecting 532 and 638 nm guided the light to a high numerical aperture apochromatic objective (60×, N.A. 1.2, water immersion, Olympus) that focused the light to a confocal volume of 1.0 fl for excitation at 532 nm and detection at 575 nm. Fluorescence from excited molecules was collected with the same objective and focused onto a 50-μm diameter pinhole. The donor and acceptor emissions were separated via a dichroic long pass filter with a dividing edge at 620 nm. Suited bandpass filters were inserted to eliminate the respective excitation wavelength and minimize spectral crosstalk. The fluorescence was detected with two avalanche photodiodes using Time-correlated Single Photon Counting with the TimeHarp 200 board. Data were stored in the Time-tagged Time-resolved Mode. Measurements were performed 25 μm deep in the solution with a total acquisition time of 1 h and repeated fresh up to four times on each protein sample (50–80 pM) in 20 mM Tris, 145 mM NaCl, 5 mM CaCl_2_ 0.01% Tween 20, pH 7.4. When specified, the thrombin-specific inhibitor argatroban was added to the solution at a final concentration of 100 μM. Signals from single molecules were observed as bursts of fluorescence. Bursts with more than 35 counts were searched with the all photon burst search (APBS) algorithm while integration time was set to 0.5 ms^43^. Appropriate correction for direct excitation of the acceptor at the donor excitation wavelength (DE = 0.15), leakage of the donor in the acceptor channel (Lk = 0.08), and the instrumental γ factor (γ = 0.85) was calculated using a mixture of double-stranded DNA models with known FRET efficiency and stoichiometry labeled with dyes AF555 and AF647^44,45^. Only molecules with a stoichiometry in the range S = 0.25–0.75 were considered in the final analysis, and their distribution was fit to Gaussian curves using Origin 2015 (OriginLab). The number of independent Gaussians was determined according to the corrected Akaike information criterion (AICc). Data recording and initial data analysis were performed using the SymphoTime Software 6.4 (PicoQuant, Berlin). Further analysis was carried out with PAM (Munich, Germany) and figures generated with Datagraph. Theoretical FRET values were obtained by coarse-grained simulations using the FPS software package^17^.

### Activation of Prothrombin

Prothrombin activation was monitored by SDS-PAGE in the presence 60 μM dansylarginine-N-(3-ethyl-1,5-pentanediyl)amine (DAPA), as detailed elsewhere^14,46^. Briefly, prothrombin (0.1 mg/ml, 1.4 μM) dissolved in 150 mM NaCl, 20 mM Tris, 5 mM CaCl_2_ was activated with FXa (0.4 nM), phospholipids (25 μM) and cofactor Va (10 nM). Following the addition of the enzyme, samples (40 μl, 4 μg) were quenched at different time intervals with 10 μl of NuPAGE LDS buffer with or without β-mercaptoethanol as the reducing agent and 20 mM EDTA. Samples were loaded into 12% SDS-polyacrylamide gels or processed by NuPAGE Novex 4–12% Bis-Tris protein gels run with MES buffer. Each experiment was repeated at least three times using three different batches of proteins. Conversion of prothrombin to thrombin was also monitored using a colorimetric assay that continuously reports the amount of thrombin that is generated upon cleavage by the prothrombinase complex. Briefly, prothrombin (250 μl, 10 nM) activation reacted with 2.5 pM factor Xa, 20 μM phospholipids, and 10 nM cofactor Va, and 24 μM chromogenic substrate FPF-pNA. Data were collected on a SpectraMax i3x Multi-Mode Detection Platform and analyzed with Origin 2015 (OriginLab Corp., Northampton, MA)^14^.

### Accession codes and data availability

Atomic Coordinates and structure factors for the proTCC structure have been deposited in the Protein Data Bank under accession code PDB ID 6BJR and 6C2W. All reagents and relevant data are available from the authors upon request. Please contact nicola.pozzi@health.slu.edu.

## Electronic supplementary material

Supplementary Information
