# Supplementary material for: Structure of prothrombin in the closed form reveals new details on the mechanism of activation
Source: Sci Rep. 2018 Feb 13;8:2945. doi: 10.1038/s41598-018-21304-1 (PMC5811608; doi:10.1038/s41598-018-21304-1)
Supplement: Supplementary file 1 — Supplementary Information [file 41598_2018_21304_MOESM1_ESM.pdf]

Supplementary Information

Structure of prothrombin in the closed form reveals new details on the mechanism of activation

Mathivanan Chinnaraj[1], Zhiwei Chen[1], Leslie A. Pelc[1], Zachary Grese[1], Dominika Bystranowska[2], Enrico Di Cera[1,]* and Nicola Pozzi[1,]*

[1]Edward A. Doisy Department of Biochemistry and Molecular Biology, Saint Louis University School of Medicine, St. Louis, MO 63104; [2]Department of Biochemistry, Wroclaw University of Science and Technology, Wybrzeze Wyspianskiego 27, 50-370 Wroclaw, Poland.

*Corresponding authors:
Nicola Pozzi
Department of Biochemistry and Molecular Biology
Saint Louis University School of Medicine
St. Louis, MO 63104
Tel: (314) 977-9257, Fax: (314) 977-9206
E-email: nicola.pozzi@health.slu.edu

Enrico Di Cera
Department of Biochemistry and Molecular Biology
Saint Louis University School of Medicine
St. Louis, MO 63104
Tel: (314) 977-9201
E-email: enrico.dicera@health.slu.edu

Running title: Crystal structure of human full-length prothrombin in the closed conformation

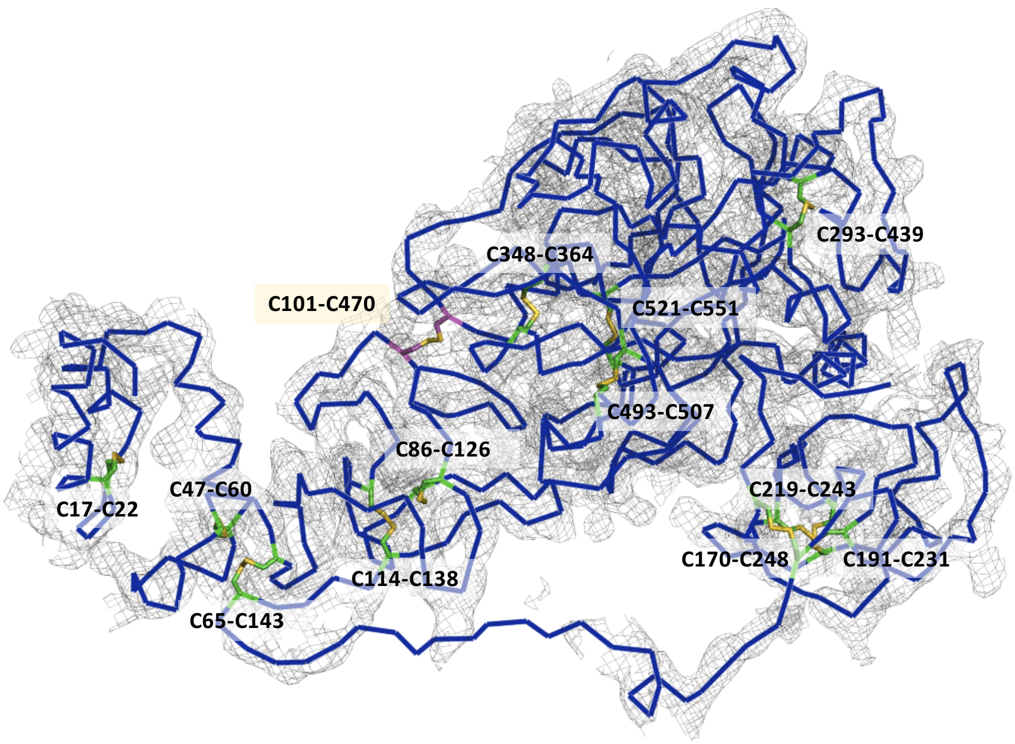

Figure 1S. Overall structure of proTCC solved at 4.1 Å resolution shown as ribbon (blue) highlighting the position of native (green stick) and engineered (magenta stick) disulphide bonds. The electron density 2Fo-Fc map is countered at 1.5σ.

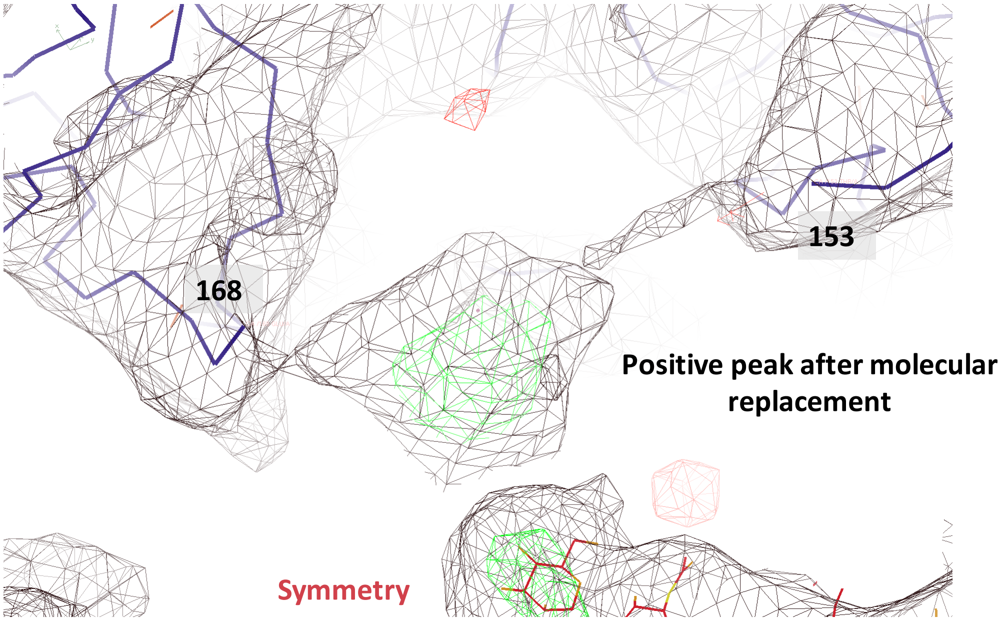

Figure 2S. Extra electron density for the Lnk2 region obtained from molecular replacement. The electron density Fo-Fc map is shown in green. The electron density 2Fo-Fc map is shown in black.

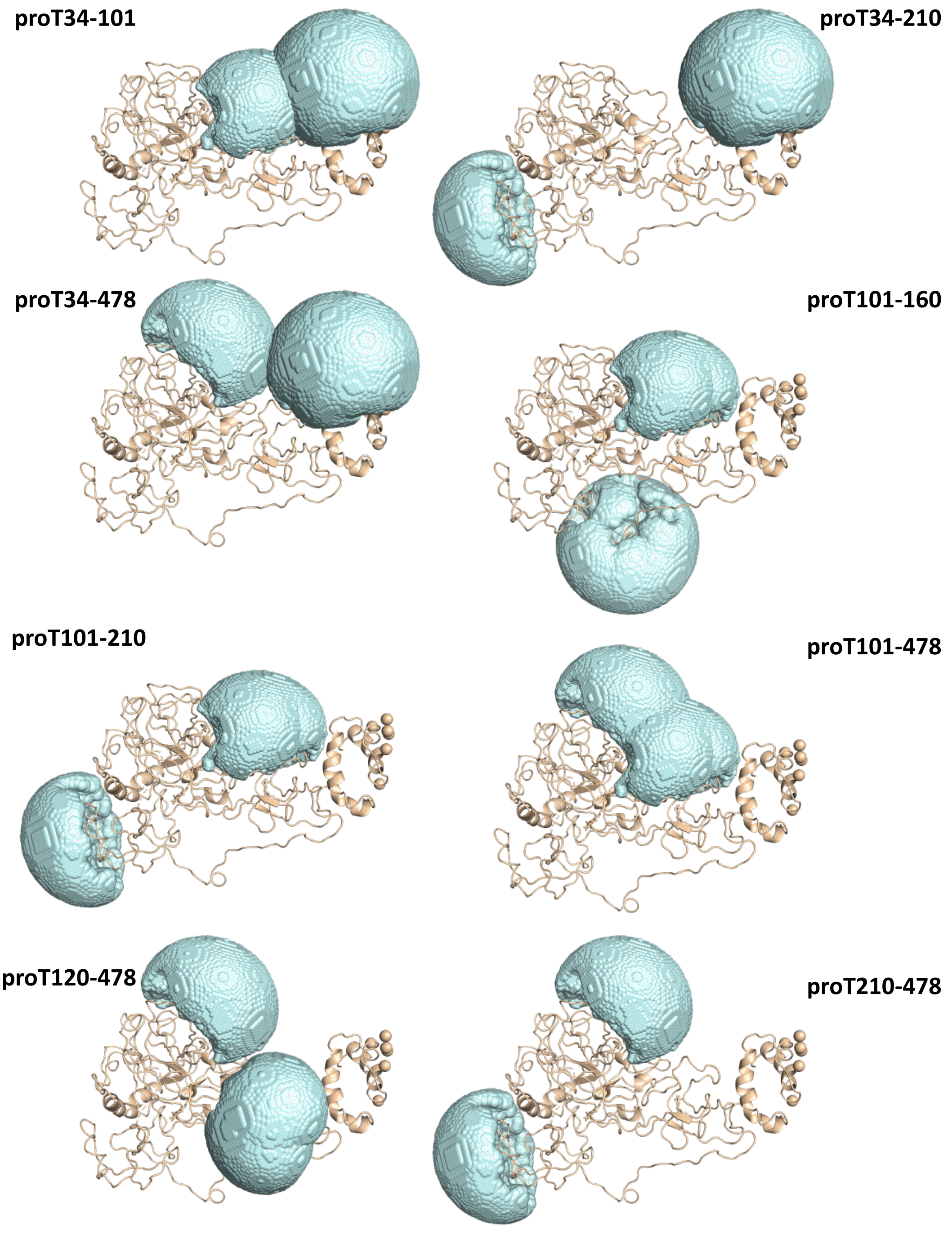

Figure 3S. Accessible Volume (AV) clouds of the fluorescent dyes attached at the sulfur atom of the engineered cysteine via a flexible linker. The calculated FRET efficiency for each FRET pair is reported in Table 2 of the main text, considering a Förster radius of 51Å. A detailed protocol for the simulations is provided by Kalinin, S. et al.[1]

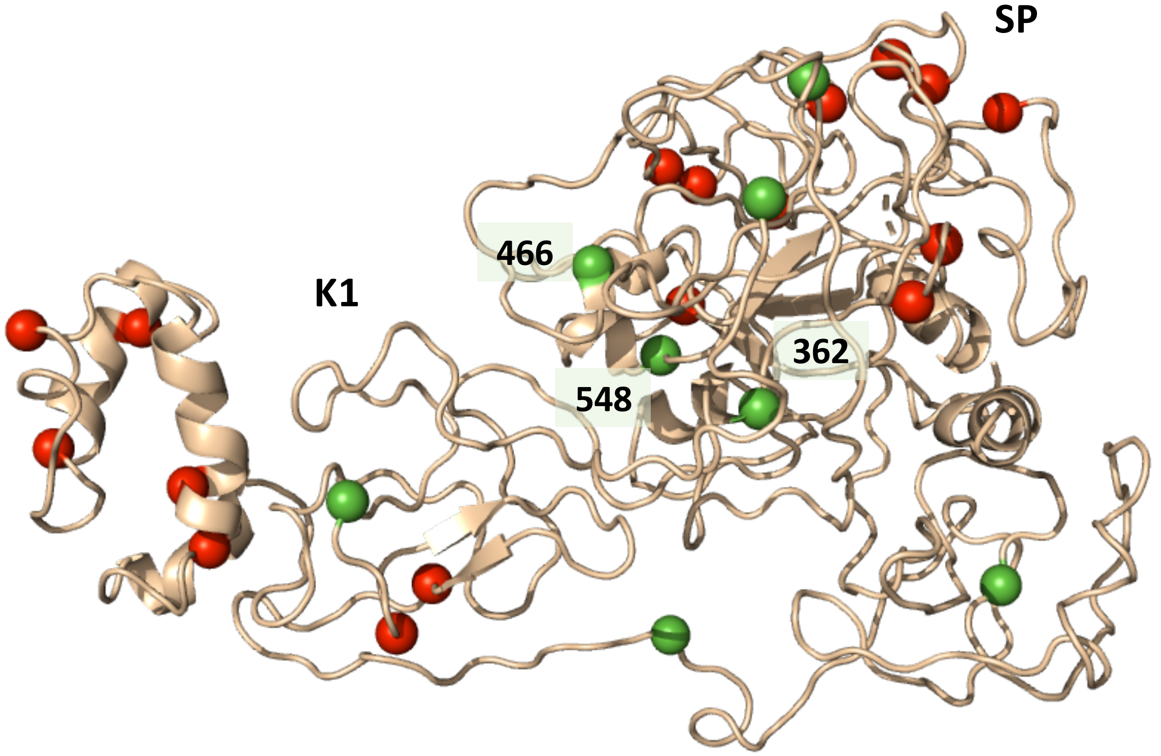

Figure 4S. Naturally occurring mutations mapped on the closed conformation of prothrombin. Red spheres indicate substitutions associated with severe bleeding. Green spheres indicate substitutions associated with mild bleeding. Ala362Thr (Vellore 1)[2], Glu466Ala (Salakta)[3], and Gly548Ala (Perija)[4] have been associated with a mild bleeding phenotype in individuals and may perturb the intramolecular interface.

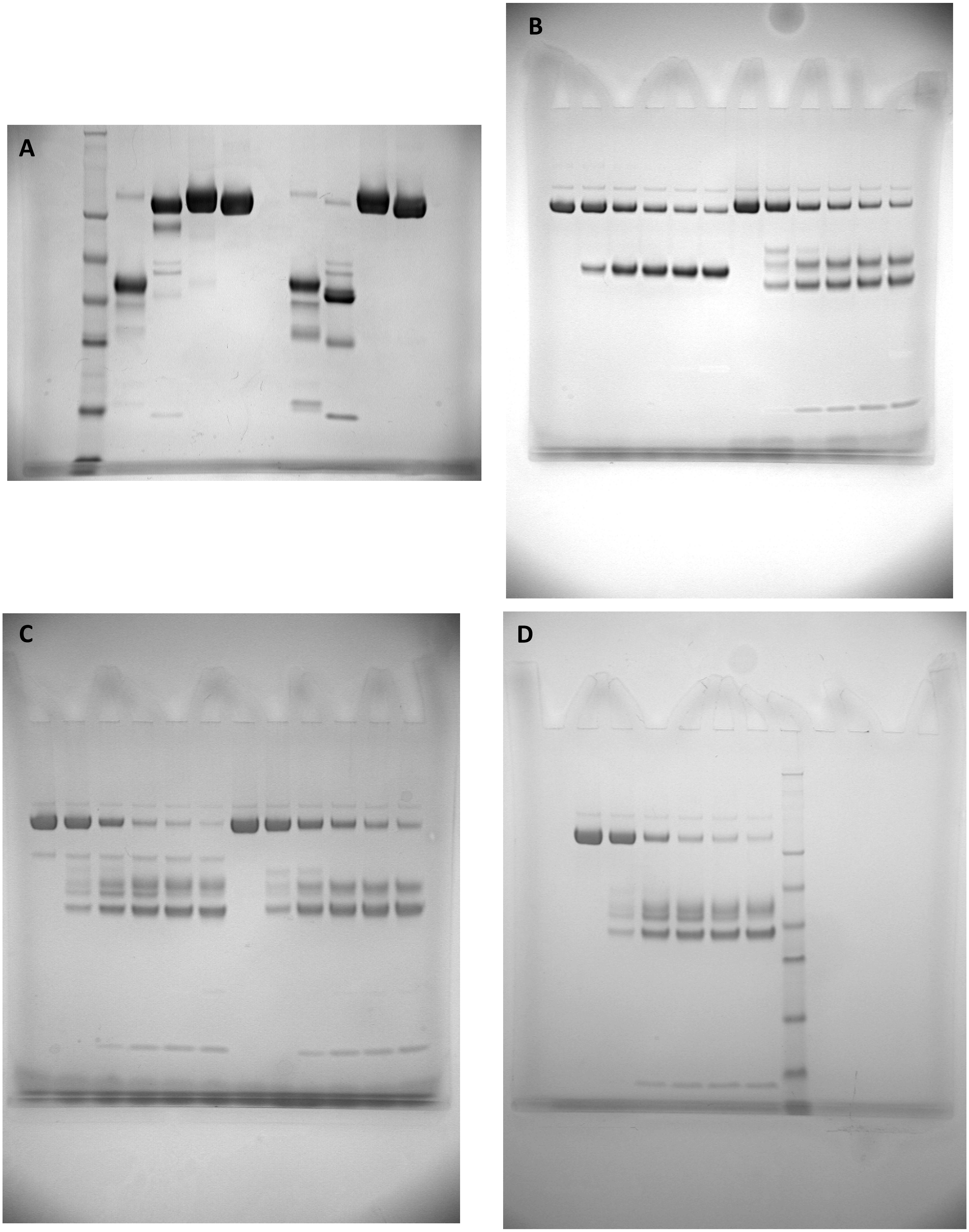

Figure 5S. Original/uncropped gels. (A) Fig 1b. (B) Right panel, proTWT in Fig 5c. (C) Right panel, proT101-470 in Fig 5c. (D) proTY93A in Fig.5c.

1. Kalinin, S. et al. A toolkit and benchmark study for FRET-restrained high-precision structural modeling. *Nat Methods* **9**, 1218-25 (2012).
2. Jayandharan, G. et al. Molecular genetics of hereditary prothrombin deficiency in Indian patients: identification of a novel Ala362 --> Thr (Prothrombin Vellore 1) mutation. *J Thromb Haemost* **3**, 1446-53 (2005).
3. Miyata, T. et al. Prothrombin Salakta: substitution of glutamic acid-466 by alanine reduces the fibrinogen clotting activity and the esterase activity. *Biochemistry* **31**, 7457-62 (1992).
4. Sekine, O. et al. Substitution of Gly-548 to Ala in the substrate binding pocket of prothrombin Perija leads to the loss of thrombin proteolytic activity. *Thromb Haemost* **87**, 282-7 (2002).
